# Evidence for a dyadic motor plan in joint action

**DOI:** 10.1038/s41598-018-23275-9

**Published:** 2018-03-22

**Authors:** Lucia Maria Sacheli, Elisa Arcangeli, Eraldo Paulesu

**Affiliations:** 1 Department of Psychology and Milan Center for Neuroscience (NeuroMi), University of Milano-Bicocca, 20126 Milan, Italy; 2 IRCCS Istituto Ortopedico Galeazzi, 20161 Milan, Italy

## Abstract

What mechanisms distinguish interactive from non-interactive actions? To answer this question we tested participants while they took turns playing music with a virtual partner: in the interactive joint action condition, the participants played a melody together with their partner by grasping (C note) or pressing (G note) a cube-shaped instrument, alternating in playing one note each. In the non-interactive control condition, players’ behavior was not guided by a shared melody, so that the partner’s actions and notes were irrelevant to the participant. In both conditions, the participant’s and partner’s actions were physically congruent (e.g., grasp-grasp) or incongruent (e.g., grasp-point), and the partner’s association between actions and notes was coherent with the participant’s or reversed. Performance in the non-interactive condition was only affected by physical incongruence, whereas joint action was only affected when the partner’s action-note associations were reversed. This shows that task interactivity shapes the sensorimotor coding of others’ behaviors, and that joint action is based on active prediction of the partner’s action effects rather than on passive action imitation. We suggest that such predictions are based on Dyadic Motor Plans that represent both the agent’s and the partner’s contributions to the interaction goal, like playing a melody together.

## Introduction

Which cognitive mechanisms allow two pianists to smoothly coordinate while playing piano four-hands? Somehow, they reciprocally keep track of what each is doing, in a holistic sense, in relation to the melody they are playing, and they adapt to each other to create music. Yet how such music can arise from the coordination of two independent brains and bodies is not fully understood. More generally, the cognitive mechanisms underlying coordination during motor interactions remain elusive, making it difficult to reproduce them by artificial agents, for example.

Joint actions (JAs), which we refer to here, are activities involving two or more agents who coordinate their plans of action to achieve an outcome in the environment^1^. They can be considered an experimental test-case to study “interactions” in general by investigating their underlying motor mechanisms, regardless of verbal exchanges. As JAs require fine-tuned interpersonal coordination in time and space, it is intuitive to hypothesize that the partner’s actions need to be somehow represented by the agent so that the agent can select an appropriate and timely response.

*Motor simulation* has repeatedly been indicated as the process that might support such representation of the partner’s behavior to promote interpersonal coordination^2–5^. Converging evidence from cognitive psychology and neurophysiology suggests that action perception and execution are tightly linked and that they rely on a “shared representational system”^6,7^ that allows for the internal simulation of an observed action in one’s sensorimotor system. Such simulation can occur outside an agent’s awareness, as when the listener mimics the speaker’s postures and gestures during a conversation, for instance^8^. Laboratory experiments have shown that observation of hand movements facilitates performing the same (congruent) as compared to a different (incongruent) action^9,10^. This effect has been attributed to the automatic (unwanted) simulation of an observed action: when an action different from the observed action is required, automatic simulation needs to be controlled by top-down processes (i.e., possibly inhibited^11,12^) that incur additional computational costs and thus induce a “visuomotor interference” effect^13^.

In short, it has been suggested that observation of others’ actions automatically triggers action simulation (due to bottom-up visuomotor associations^10^), whereas top-down, rule-based associations are enlisted to perform a motor response that differs from the observed one (*Dual-Route* hypothesis, see^14^, but also similar accounts^11,12^). This view is supported by evidence in patients with frontal lesions. Having lost the ability to top-down control automatic behaviors, they show signs of unwanted imitation of observed actions^15^ that correlate with impairment on more social tasks that measure the ability to understand others’ mental states^16^.

**Figure 1:**
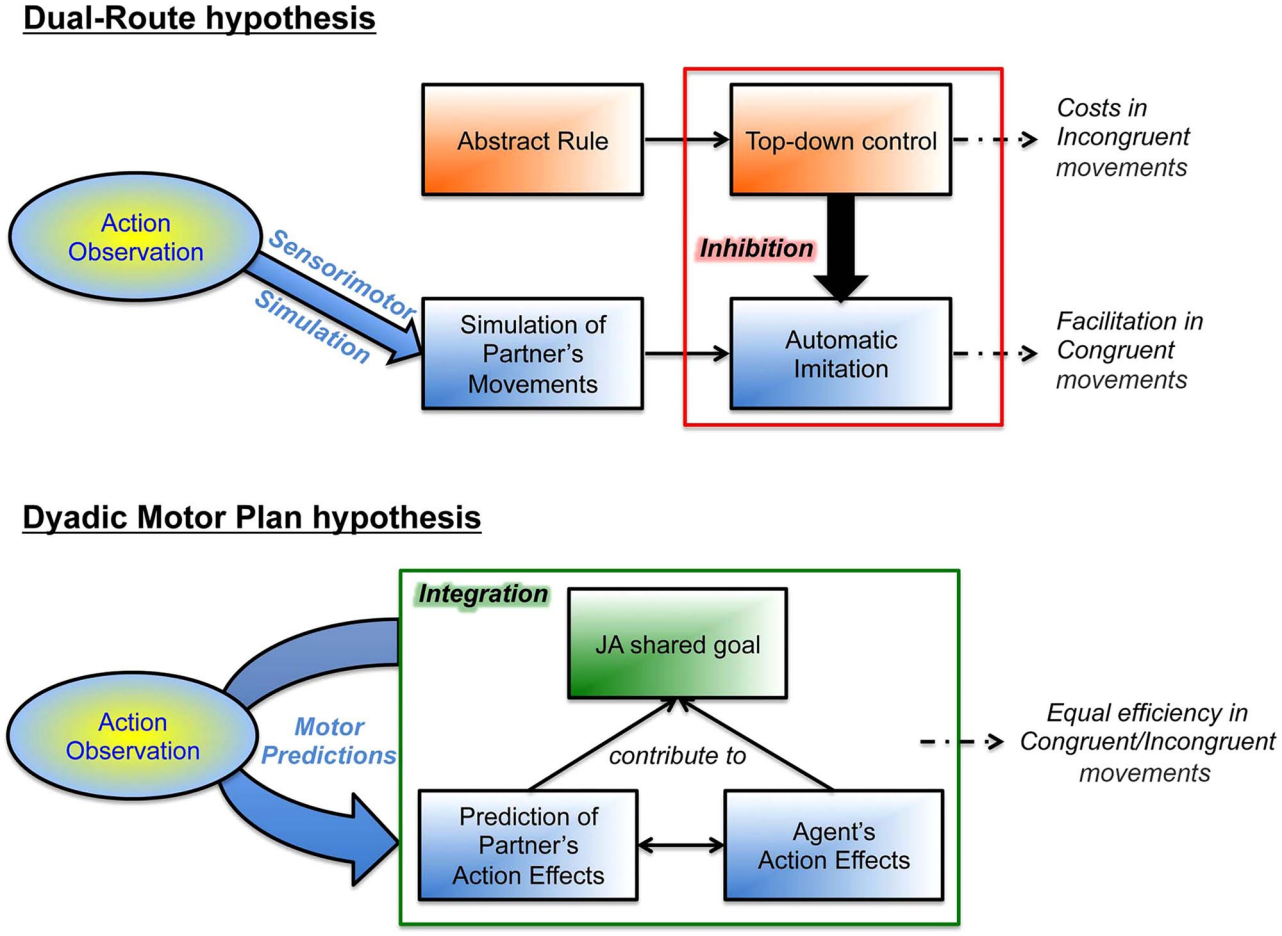
Scheme illustrating the Dual-Route and the Dyadic Motor Plan hypotheses and the expectations about how the agent’s performance is modulated in congruent (imitative) vs. incongruent (non-imitative) actions.

Although simulation of a partner’s action might be beneficial in such social circumstances as observational learning, it may be detrimental when the required interactive response is physically incongruent with the observed action, as when handing over and receiving an object. In this latter case, the Dual-Route hypothesis postulates that “shared representations” need to be kept under control to avoid that we imitate others all the time, and that such control implies computational costs in terms of visuomotor interference (Fig. 1). Everyday life and experimental evidence^17–19^ show, however, that interactions requiring incongruent responses are not necessarily more demanding than imitative ones. Moreover, during motor interactions, agents do not usually coordinate with one another according to their reciprocal actions but rather by their action effects. Musicians playing a piano-violin duet integrate their movements with the melody rather with the movements themselves: one might thus argue that what is relevant for interpersonal coordination is not the specific action that a partner performs but rather its effects in the environment (e.g., the notes that it is producing). Indeed, a partner’s action effect constitutes his/her contribution to the achievement of the JA goal (e.g., playing a melody), and it might be what agents take into account and adapt to during the JA^20,21^, see^22^.

This leads to suggest an alternative hypothesis: motor interactions might be based on the ability to integrate one’s own and a partner’s action within a unitary, dual-person (dyadic) motor plan that incorporates the goal of the JA that both partners share. This *Dyadic Motor Plan* would allow an agent to represent (and predict) the effects of a partner’s actions: agents select an appropriate response based on (prediction of) such effects according to the overarching JA goal, without requiring top-down inhibition of sensorimotor simulation (Fig. 1). The aim of this study was to directly compare the Dual-Route and the Dyadic Motor Plan hypotheses to characterize the cognitive processes that underpin motor interactions.

In our novel paradigm, participants played music in turn with a partner by performing two actions on a cube-shaped response box (grasping the sides vs. pressing the top) that generated two different tones (C note and G note). In the interactive, joint action (JA) condition, the participants shared with their partner the goal of playing a pre-learned four-note melody by alternating playing one note each; in the control, non-interactive (Non-Int) condition, the participants’ and their partners’ actions and tones were unrelated, and the participants were cued which pair of notes to play in two consecutive trials, independent of the notes their partner played. The participants were unaware that, in both conditions, 50% of the trials required either physically congruent or incongruent movements. In a second experiment, the partner’s action-effect association was manipulated, so that the association between the partner’s action (grasping vs. pointing) and the ensuing effects (C note vs. G note) was reversed in 50% of the trials, while it never changed for the participants. The comparison between physically congruent and incongruent actions measures a participant’s tendency to simulate and involuntarily imitate the observed action (as indexed by the emergence of visuomotor interference). Differently, in the trials in which the partner’s action-effect association is reversed the potential interference measures the participant’s tendency to predict the effects of the partner’s actions from observation: indeed, if agents try to actively predict the effects of an observed action based on their own action-effect associations, they might show signs of prediction error when such associations are reversed in the partner.

We had the following expectations. The *Dual-Route* hypothesis (see^14^, but also^11,12^) suggests that any observed action automatically triggers action simulation in the observer’s motor system, and that top-down inhibition is enlisted to keep such simulation under control. This account predicts a cost of performance efficiency if an incongruent motor response is required (i.e., it predicts visuomotor interference), independent of the degree of task interactivity. In contrast, our *Dyadic Motor Plan* hypothesis suggests that visuomotor interference emerges only in non-interactive contexts, when the action is passively observed by, and it is irrelevant for, the onlooker. During an interaction, the presence of an interaction goal allows both partners to activate motor plans describing each agent’s contribution to achievement of the interaction goal, and the agents show signs of anticipatory simulation of the partner’s action effect (which is expected to be aimed at achieving the interaction goal) that would be selective for interactive contexts and independent of action (in) congruence (Fig. 1).

A third and fourth experiment tested the robustness of our results and provided a replication in independent samples (see Supplementary Information).

## Results

**Figure 2:**
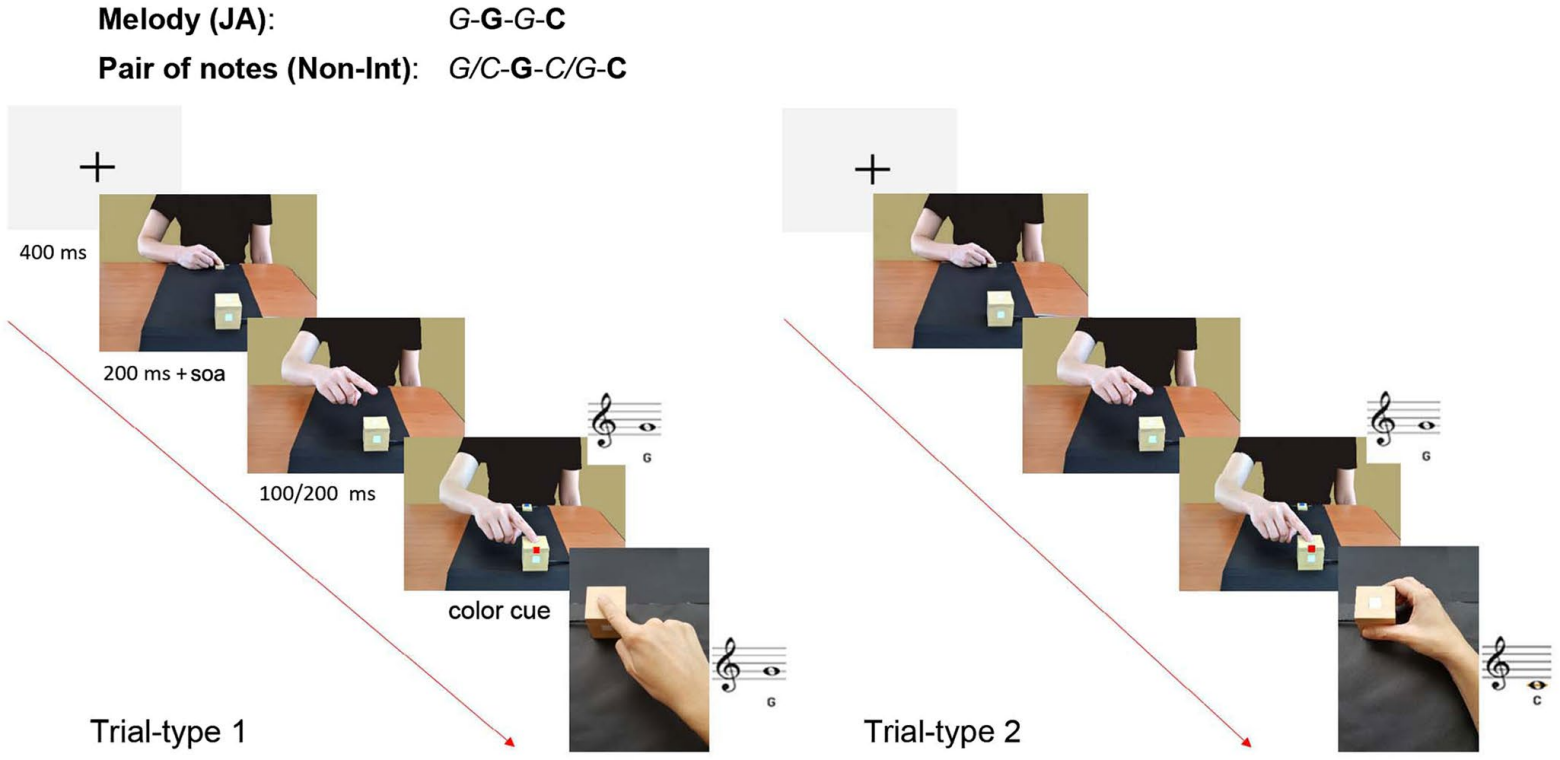
The trial timeline of the Non-Interactive (Non-Int) and the Joint Action (JA) conditions was identical in all experiments. All experiments included the factor Trial-type because the color cue directed participants on what to do in two consecutive trials. Task performance in Trial-type2 was always more efficient than in Trial-type1, independent of other experimental manipulations. The color cues in the JA and the Non-Int conditions convey the same amount of information regarding the action that the participant has to perform in two consecutive trials (e.g., play a G and then a C). While the color cue in the JA condition also informs the participants about what their partner will do, in the Non-Int condition the partner’s action is irrelevant and so not specified. The trial sequence on the left (Trial-type 1) is a congruent trial and the one on the right (Trial-type 2) is an incongruent trial. SOA denotes stimulus onset asynchrony (range 100 to 700 ms).

In all experiments, each musical sequence (either melodies in the JA condition or pairs of notes in the Non-Int condition) was divided in two trials of different “type”, Trial-type1 and Trial-type2, because the instructions (provided via color cues) informed participants on what to do in two consecutive trials (see Methods and Fig. 2): in Trial-type1, they observed their partner’s action before being cued which note they had to play, whereas in Trial-type2 they already knew what to do before observing their partner’s action because they had already seen the cue in the preceding trial.

Performance in Trial-type2 was expected to be more efficient than in Trial-type1 as the participants were able to plan their response in advance. The factor Trial-type (Trial-type1 vs. Trial-type2) was included in the design of all experiments, although it was not crucial for the purpose of the study. For all experiments, we analyzed inverse efficiency scores (IES), i.e., the response times (RTs)/ACC ratio, to appropriately weigh the impact of speed and accuracy and thus capture participant performance as a whole^23^.

### Experiment 1 – Physical congruence of action does not modulate behavior in JA

In Experiment 1 (N = 25) we tested whether the emergence of visuomotor interference (defined as a computational cost in incongruent vs. congruent condition) is modulated by the interactive nature of the task. Analyses were based on a 2 × 2 × 2 repeated-measures design with Task (JA vs. Non-Int) x Trial-type (Type1 vs. Type2) x Congruence of Actions (Congruent vs. Incongruent) as within-subject factors.

**Figure 3:**
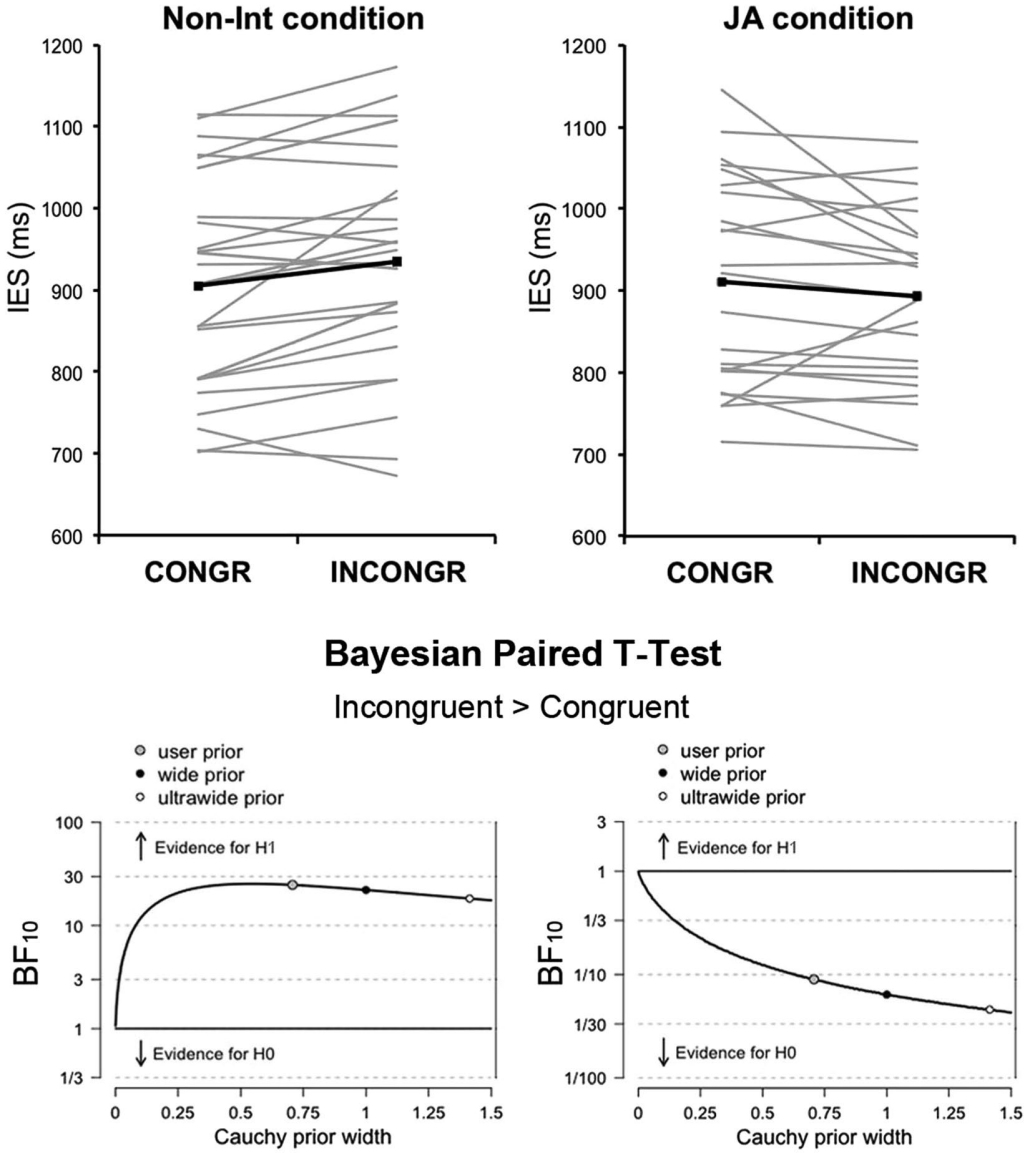
Difference in inverse efficiency scores (IES) in Experiment 1. Task x Congruence of Action interaction (upper panel): the grey lines indicate single-subject data and the thick black lines indicate the mean values. Overall performance on the Joint Action (JA) task was as efficient as performance on the Non-Interactive (Non-Int) task, as suggested by the absence of a significant main effect of Task in the ANOVA. The Bayes Factor Robustness Check as implemented in JASP (lower panel): given the data, Experiment 1 provides strong evidence (BF_10_ > 10) supporting a significant “IES-Incongruent > IES-Congruent” effect in the Non-Int condition, and strong evidence in favor of the null hypothesis (i.e., absence of the IES-Incongruent > IES-Congruent effect, BF_10_ < 0.10) in the JA condition.

We found a significant main effect of Trial-type (F(1,24) = 166.18, *p* < 0.001, η_p_^2^ = 0.87), indicating that performance in Trial-type2 was always more efficient (i.e., IES were lower) than in Trial-type1. More importantly, there was a significant Task x Congruence interaction (F(1,24) = 7.96, *p* = 0.009, η_p_^2^ = 0.25), indicating that performance in Incongruent actions was less efficient (i.e., IES were higher) than in Congruent actions selectively on the Non-Int task (*p*_corr_ = 0.01) but not on the JA task (*p*_corr_ = 0.24), suggesting the presence of visuomotor interference only in the former (Fig. 3). These results indicate that the Dual-Route hypothesis, which postulates a computational cost in Incongruent vs. Congruent actions, holds only in the Non-Int but not in the JA condition.

To gain more explicit evidence for the lack of an effect of Congruence in the JA condition, we applied a Bayesian approach and tested, separately for the JA and the Non-Int condition, the strength of the evidence in favor of the presence of a visuomotor interference effect (i.e., IES in Incongruent > IES in Congruent condition). The Bayesian paired-samples t-test showed strong evidence supporting the alternative hypothesis (i.e., presence of a visuomotor interference, BF_10_ = 24.75) for the Non-Int task, and strong evidence supporting the null hypothesis (i.e., absence of visuomotor interference, BF_10_ = 0.09) for the JA task.

### Experiment 2 – Prediction of action effects uniquely takes place in interactive contexts

**Figure 4:**
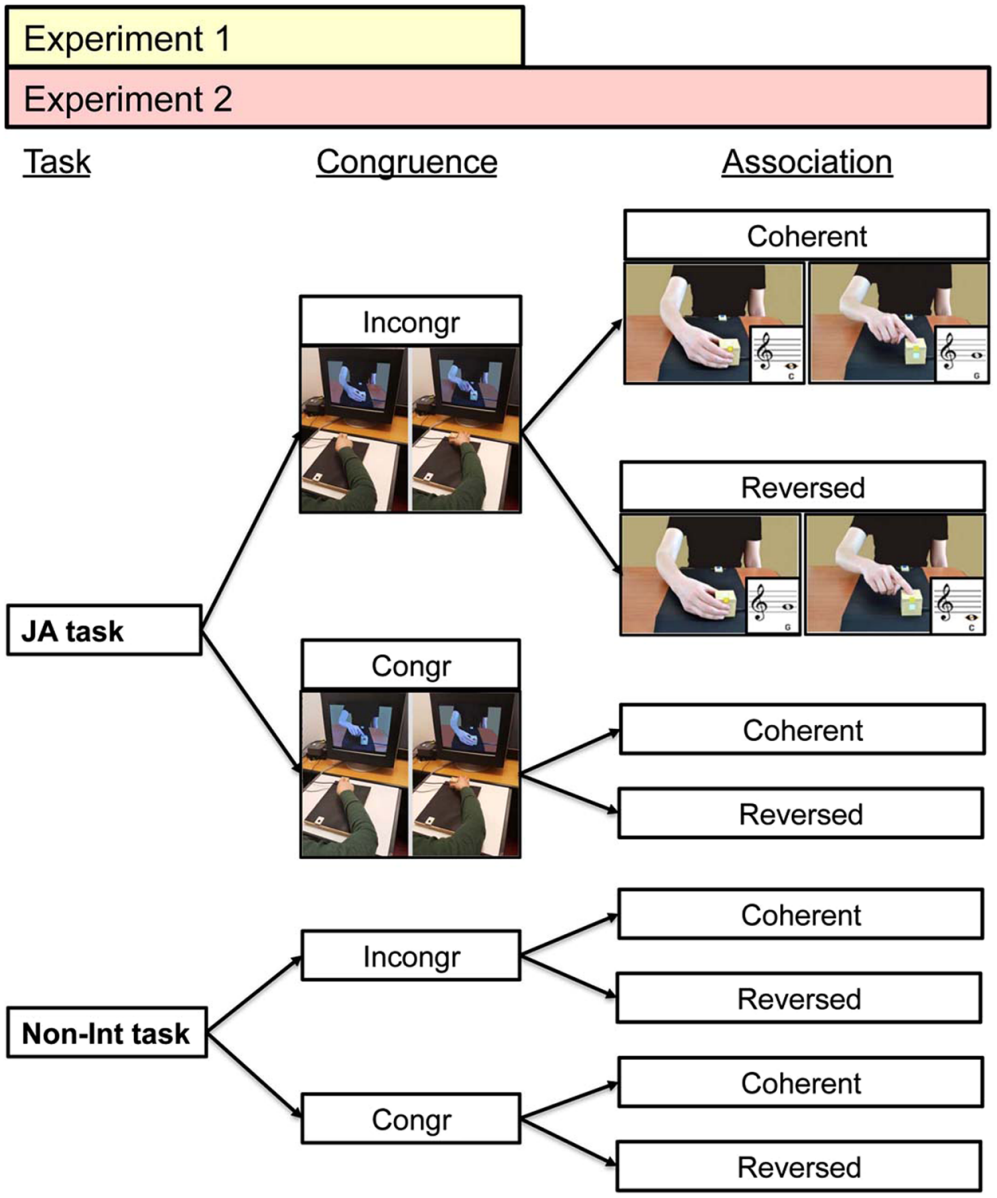
Schematic illustration of the experimental design in Experiment 1 (2 × 2 × 2 design: Task x Trial-type x Congruence of Actions) and Experiment 2 (2 × 2 × 2 × 2 design: Task x Trial-type x Congruence of Actions x Action-Note Association).

Experiment 2 (N = 23) was designed to replicate the results from Experiment 1 and to investigate the role of predictions about the partner’s action effects. Analyses were based on a 2 × 2 × 2 × 2 repeated-measures design with Task (JA vs. Non-Int) x Trial-type (Trial-type1 vs. Trial-type2) x Congruence of Actions (Congruent vs. Incongruent) x Partner’s Action-Note Association (Coherent vs. Reversed as compared to the participant’s) as within-subject factors (see Fig. 4).

Analysis of IES showed a significant Task x Congruence interaction (F(1,22) = 9.69, *p* = 0.005, η_p_^2^ = 0.30) and Task x Association (F(1,22) = 31.20, *p* < 0.001, η_p_^2^ = 0.58) interactions, indicating that both the effect of physical Congruence of Actions and of the Partner’s Action-Note Associations were modulated by Task interactivity, as predicted by the Dyadic Motor Plan hypothesis. However, the ANOVA also showed significant main effects and interactions up to the quadruple Task x Trial-type x Congruence x Association interaction (Table 1).

**Table 1:** ANOVA of inverse efficiency scores (IES) of Experiment 2.

Effect	F	df	*p*	η^2^_p_
Main effect of Task	21.14	1,22	<0.001	0.49
Main effect of Trial-type	205.09	1,22	<0.001	0.90
Main effect of Association	25.96	1,22	<0.001	0.54
Task x Trial-type	54.24	1,22	<0.001	0.71
***Task x Association***	***31.20***	***1,22***	<***0.001***	***0.58***
***Task x Congruence***	***9.69***	***1,22***	***0.005***	***0.30***
Trial-type x Association	12.46	1,22	0.001	0.36
Association x Congruence	5.89	1,22	0.02	0.21
Task x Trial-type x Association	13.93	1,22	0.001	0.39
Task x Trial-type x Congruence	5.23	1,22	0.03	0.19
Task x Association x Congruence	5.17	1,22	0.03	0.19
Trial-type x Association x Congruence	7.15	1,22	0.01	0.24
Task x Trial-type x Association x Congruence	7.37	1,22	0.01	0.25

The expected Task x Congruence and Task x Association interactions are given in *bold and italics*.

To make these higher-level interactions easier to interpret, we performed follow-up ANOVAs separately for the Non-Int and the JA tasks. The ANOVA of the Non-Int task showed a significant main effect of Trial-type (F(1,22) = 142.63, *p* < 0.001, η_p_^2^ = 0.86), indicating that performance in Trial-type2 was more efficient (i.e., IES were lower) than in Trial-type1, and a significant main effect of Congruence (F(1,22) = 13.29, *p* < 0.001, η_p_^2^ = 0.37), indicating that Incongruent actions were less efficient than Congruent actions, demonstrating the presence of visuomotor interference. No other main effect or interaction was significant (all *p*s > 0.3), including the main effect of Association (*p* = 0.60). The ANOVA of the JA task revealed that all main effects and interactions were significant up to the Trial-type x Congruence x Association triple interaction (F(1,22) = 7.39, *p* = 0.012, η_p_^2^ = 0.25) (Table 2).

**Table 2:** Follow-up ANOVA of inverse efficiency scores (IES) in the Joint Action condition of Experiment 2.

Effect	F	df	*p*	η^2^_p_
Main effect of Trial-type	154.26	1,22	<0.001	0.87
Main effect of Association	29.45	1,22	<0.001	0.57
Main effect of Congruence	5.97	1,22	0.023	0.21
Trial-type x Association	13.60	1,22	0.001	0.38
Trial-type x Congruence	4.72	1,22	0.040	0.18
Association x Congruence	5.61	1,22	0.027	0.20
Trial-type x Association x Congruence	7.39	1,22	0.012	0.25

**Figure 5:**
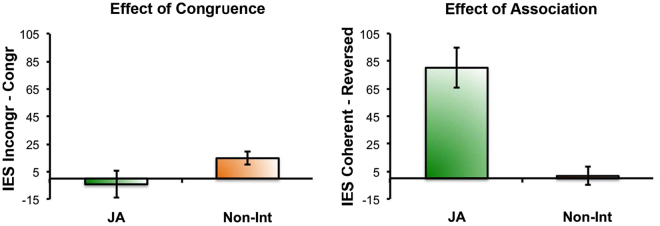
On the left, Effect of Congruence of Action in the Joint Action (JA) and the Non-Interactive (Non-Int) tasks in Trial-type2 data in Experiment 2: the effect is plotted as the mean of individual differences “IES-Incongruent minus IES-Congruent trials”. On the right, the effect of Partner’s Action-Note Association in Trial-type2 data in Experiment 2: the effect is plotted as the mean of individual differences “IES-Reversed minus IES-Coherent Association trials”. Error bars indicate standard error of the mean [SEM].

We performed follow-up ANOVAs separately for Trial-type1 and Trial-type2. Here we focus on the key findings of the analysis of data collected in Trial-type2. Follow-up ANOVA of Trial-type2 data only showed a significant main effect of Association (F(1,22) = 30.95, *p* < 0.001, η_p_^2^ = 0.58), indicating that participant performance was less efficient (i.e., IES were higher) when interacting with a partner who played with a reversed action-note association. No other main effect or interaction was significant (all *p*s > 0.4), including the main effect of physical Congruence of Actions (*p* = 0.67). See Fig. 5.

As a whole, follow-up ANOVA of Trial-type1 data was consistent with these results: the analysis showed a main effect of Association and a pattern incompatible with the presence of visuomotor interference, thus contradicting what would be predicted by the Dual-Route hypothesis and providing further evidence supporting the Dyadic Motor Plan hypothesis (see Supplementary Information for more details).

Overall, the results of Experiment 2 showed that performance on the Non-Int task was influenced by the physical congruence of actions independent of the partner’s action-note association: this confirms the results of Experiment 1 and suggests that the participants tended to involuntarily imitate their partner’s action in the Non-Int condition. Conversely, participant performance on the perceptually-matched JA task showed no signs of visuomotor interference, while performance was less efficient when the partner’s action-note association was reversed. This suggests that the participants recruited predictive motor processes to anticipate (from observation) the effect of their partner’s action, which constitutes the partner’s contribution to achieving the JA goal (i.e., playing the melody).

We performed a Bayesian control analysis of the data from Experiment 2 to gain more explicit evidence for the lack of an effect of Association in the Non-Int condition and of Congruence in the JA condition. Bayesian paired-sample t-tests on Experiment 2 data were performed to test, separately for the JA and the Non-Int conditions, evidence in favor of the alternative hypothesis with regard to (i) the Effect of Congruence (i.e., IES in Incongruent > IES in Congruent condition), and (ii) the Effect of Association (i.e., IES in Reversed-Association > IES in Coherent-Association condition). With regard to the Effect of Congruence, there was very strong evidence supporting the alternative hypothesis (i.e., presence of visuomotor interference, BF_10_ = 51.95) in the Non-Int condition [where IES-Incongruent (889.19 ± 107.27 ms) > IES-Congruent (868.73 ± 104.88 ms)], and strong evidence for the null hypothesis (i.e., absence of visuomotor interference, BF_10_ = 0.07) in the JA condition [where IES-Incongruent = 1012.37 ± 175.00 ms; IES-Congruent = 1076 ± 200.56 ms]. With regard to the Effect of Association, there was evidence supporting the null hypothesis (i.e., absence of interference in the Reversed as compared to the Coherent Association, BF_10_ = 0.15) in the Non-Int task [where IES-Reversed = 877.36 ± 102.49 ms, IES-Coherent = 880.56 ± 109.77 ms], and very strong evidence for the alternative hypothesis (i.e., interference in the Reversed as compared to the Coherent Association, BF_10_ = 2451) in the JA condition [where IES-Reversed (1129.70 ± 224.32 ms) > IES-Coherent (958.67 ± 155.43)].

Two control experiments run on two independent samples (Experiments 3 and 4) replicated and confirmed these results (see Supplementary Information).

## Discussion

Although humans show a strong tendency to imitate others, most everyday-life interactions are based on non-imitative behaviors, like handing over and receiving an object. The aim of the present study was to provide a theoretical model describing cognitive mechanisms that characterize motor planning during motor interactions and possibly support the ease whereby non-imitative interactions occur in daily life. Ultimately, this theoretical framework might pave the way to future research on characteristic features of efficient interactions to model, for instance, cost-effective motor interactions in artificial agents.

To this end, our paradigm compared two alternative hypotheses for the cognitive bases of interactive behaviors, the Dual-Route hypothesis and the Dyadic Motor Plan hypothesis. We created two perceptually matched conditions, the Joint Action (JA) and the Non-Interactive (Non-Int) tasks, which allowed us to test, in an interactive vs. non-interactive context, the role of (i) sensorimotor simulation of a partner’s action (Experiment 1) and (ii) predictions about the partner’s action effects (Experiment 2). The Dual-Route hypothesis predicts that unwanted simulation of a partner’s action always takes place and that it needs to be top-down controlled when incongruent responses are appropriate, leading visuomotor interference to emerge. Differently, according to the Dyadic Motor Plan hypothesis, no visuomotor interference is expected in interactive contexts where predictions about the partner’s action effects would modulate the agent’s performance.

Our data support the Dyadic Motor Plan hypothesis. Indeed, in line with the Dyadic Motor Plan hypothesis, task interactivity was observed to modulate the recruitment of sensorimotor simulation, suggesting that different motor planning processes take place during the Non-Int and the JA conditions. While performance in the Non-Int condition showed signs of involuntary imitation of the partner’s action (as measured by visuomotor interference), performance in the JA condition was unaffected by physical congruence of actions but it was perturbed by the reversal of the partner’s action-effect association: this suggests that rather than passively imitating their partner’s actions, the participants tried to predict their effects from observation. When such predictions were violated, performance decayed.

The results of Experiments 1, 2, and 3 (see Supplementary Information) showed that the experimental stimuli and set-up applied in the present study *per se* resemble the one typically applied in visuomotor interference paradigms^9^. Visuomotor interference emerged in the Non-Int condition, demonstrating that our stimuli can evoke sensorimotor simulation in the observer. That such interference did not arise in the JA condition is shared by previous behavioral studies^17–19^ that reported no performance cost in joint actions requiring incongruent as compared to congruent responses. This is the first time, however, that two perceptually matched conditions are directly compared, demonstrating that the previous results were not due to differences in lower-level features of the stimuli or task instructions. Moreover, in our paradigm both the Non-Int and the JA conditions entailed the use of visually cued instructions: in both conditions the participants could, in principle, completely ignore their partner’s behavior and plan their response based on the color cue. Evidence that this was not the case, and that the participants showed an opposite effect of Congruence and Association on the two tasks, suggests that it is enough to frame a task as “interactive” to modulate the motor planning processes recruited by participants, independent of the real need for on-line interpersonal coordination required by the task itself. The results from Experiment 1 showed an identical pattern independent of Trial-type, i.e., regardless of whether the participants knew (Trial-type2) or did not know (Trial-type1) what response to give while observing their partner’s action. This latter point also suggests that our results do not depend merely on pre-learning of the melody in the JA condition: indeed, while observing their partner’s action in Trial-type1, the participants did not yet know which melody they had to play (Fig. 2), and yet they showed the same effects (i.e., no visuomotor interference and an effect of association reversal that was selective for the JA condition).

In our view, the lack of visuomotor interference in the JA condition does not indicate that the onlooker does not simulate the observed action; instead, it supports previous claims that motor simulation promotes interpersonal coordination and is strongly influenced by the agent’s own motor experience^2,3^. We propose that the motor processes at play during interactive vs. non-interactive contexts are just different in nature.

As shown by the results of Experiments 2 and 4 (see Supplementary Information), the partner’s action is not ignored (e.g., by focusing on their partner’s notes while neglecting his movements), otherwise the participants would not notice the reversal of action-note associations, as they indeed do. We argue, however, that instead of passively simulating (and involuntarily imitating) the observed action, the participants applied predictive processes to anticipate the effect of their partner’s action (i.e., his note). These predictive processes are likely motor in nature. According to Hommel^24^, motor representations entail associations between action-contingent events (action effects) and their cause (the related action) through a general process termed “feature binding”^25^. This causal link between an action effect and its related action probably forms as a result of Hebbian learning mechanisms that involve sensorimotor areas^26^; once established, they influence human and non-human primate behavior during both action perception and action execution^27–30^.

In the JA condition, agents can form expectations about their partner’s contribution based on the interaction goal (Fig. 1). Furthermore, they might apply action-effect motor associations learnt through their own motor experience to predict (from observation of their partner’s movements) the effects the partner’s action will generate. By doing so, they monitor whether the effects correspond to their expectations. This promotes interaction efficiency when the partner’s and the agent’s action-effect associations are coherent with each other (independent of physical congruence of actions), whereas it would induce a prediction error (and consequently a decay in performance) when action-effect associations differ. That this latter effect is already at play in young children (see^31^) might suggest that the underlying mechanism does not imply top-down executive control, which is still under development in preschoolers^32^. Moreover, the motor nature of Dyadic Motor Plans is suggested by the role that visuomotor parietal areas^33,34^ seem to play in non-imitative interactions. Dyadic Motor Plans require the ability to ***integrate*** motor representations of both the agent’s and the partner’s (predicted) action in a unitary motor representation. Evidence that planning simultaneous JAs might even apply bimanual models (characterized by highly integrated motor representations^35^) also supports this interpretation.

Our results are in line with previous suggestions that JA partners form representations that specify the interaction outcomes^36–38^; however, we show for the first time that motor interactions lead to a shift from an involuntary simulation of the observed action (characterizing non-interactive contexts) to the active prediction of action effects. In JA, the motor system is “prepared” to predict - from minimal kinematic cues - which effect the partner’s action will have (e.g., the note it will generate) and to rapidly associate it with the correct response (e.g., the next note of the melody) depending on the overarching interaction goal (e.g., the melody itself). Therefore, awareness of the interaction goal, e.g., knowledge about the melody, creates a link between the partner’s and the agent’s notes. We define Dyadic Motor Plan as a motor representation that links what the agent and the partner need to do to achieve the JA goal.

Since the recruitment of predictive motor processes strictly depends on the activation of a Dyadic Motor Plan and knowledge about the interaction goal, it is clear why prediction was not in place in the Non-Interactive task (as suggested by the lack of an effect of Association): anticipating the note the partner is about to play only makes sense if it helps to prepare what to do next. We suggest that “interactive” motor planning is characterized by a hierarchical structure where each partner’s contribution (e.g., expected action effects) is conditional to the interaction goal^39,40^. It has been argued that individual goals shape the coordination of muscular synergies during motor planning of individual actions^41^ and that the same motor processes allow a person to understand another person’s goals by observing their movement kinematics, thanks to reversed inference^26,42,43^. We suggest that, in similar fashion, interaction goals organize co-agent behaviors by shaping “interpersonal motor synergies”^44^. In other words, just as predictions about individual goals bias one’s perception of others’ actions^45,46^, knowledge about the interaction goal might generate strong inferences as to what one’s partner will do. This would promote interaction efficiency in most cases, albeit exposing the agent to making prediction errors, as seen in the Reversed-Association condition.

Within this framework, becoming an efficient interaction partner depends on the ability to develop (through motor experience) efficient Dyadic Motor Plans that enable agents to quickly infer their partner’s next move and to appropriately plan a response. From an information technology perspective, evidence that robots become more “collaborative” when performing an interactive task via the implementation of plans that incorporate both the robot’s and the human agent’s action flows (using a Human Aware Task Planner^47^ see^48,49^) is within this line of reasoning. Our framework suggests that artificial agents need to be equipped with a computational model that reflects the hierarchical structure of the Dyadic Motor Plan, which derives individual sub-goals from interaction goals. From a developmental perspective, what children need to learn to become efficient interactive partners might not be so much related to “social” skills such as perspective taking or understanding others’ minds (see for instance^50^), but rather may be based on motor planning skills like the ability to represent Dyadic Motor Plans and act accordingly during an interaction. This is an area for future research.

To return to our initial question about the cognitive mechanisms that allow pianists to smoothly coordinate when playing the piano four-hands, we suggest that it is the ability, developed in decades of practice, to integrate their own and the other pianist’s “playing” actions within a unitary Dyadic Motor Plan that incorporates the interaction goal (i.e., making music). While our analytical and minimalistic approach cannot capture the overall phenomenological experience of interactive players, the Dyadic Motor Plan, embodied in very simple gestures, may represent a scalable building block that could be applied to different dimensions of human interactions.

## Methods

The experimental protocol was approved by the ethics committee of the University of Milano-Bicocca (Italy) and was carried out according to the ethical standards of the 1964 Declaration of Helsinki and later amendments. All participants gave their written, informed consent to take part in the study in exchange for course credits and were debriefed as to the purpose of the study at the end of the experimental procedures. Professional musicians were not recruited.

### Experiment 1

#### Participants

A total of 25 participants took part in the study (10 men, age range 20–27 years, mean 23.8 ± 1.68). All participants were right-handed as confirmed by the Edinburgh Handedness Inventory^51^, reported normal or corrected-to-normal vision, and were naive as to the purpose of the experiment.

#### Stimuli and apparatus

We suggest that readers view the Supplementary Videos to gain a clear idea of the experimental set-up and procedure. Participants were comfortably seated at a rectangular (60 × 110 cm) table and watched a LCD monitor (1024 × 768 resolution) at a distance of ~60 cm from their eyes. The response device (BrainTrends ltd) consisted of a custom-made 5-cm wooden cube placed 40 cm to the front and 4.5 cm to the left of the midline. Touch-time on the cube was recorded by activating touch-sensitive buttons (1 cm wide), one located on the top and two on the sides. Before each trial, participants positioned their right hand with index finger and thumb gently opposed over a start-button (2 × 1 cm) located 28 cm from the cube and 4.5 cm to the left of the midline. They were instructed to either press the top button on the cube with their index finger (pointing action) or to press the side buttons with their thumb and index fingers (grasping action). Pressing the top button generated a C note (~261 Hz) and pressing the side buttons generated a G note (~392 Hz). The two sounds had the same intensity (4 dB) and duration (100 ms). A third, raspberry-like sound (duration 100 ms) was emitted as an error signal. Auditory feedback was via headphones.

The participants responded to visual stimuli that differed during the course of the experiment: small colored squares appeared on the computer screen during the Learning phase (see Supplementary Information), while during the Test phase (see below) a virtual partner was displayed in different positions (Fig. 2): (i) starting-position, (ii) implied motion posture (depicting the pointing/grasping actions at mid-flight), and (iii) a final position (depicting the end of the pointing/grasping action). The final-position image included a small colored square at the center of the partner’s cube that gave the color-cued instructions for the playing melody/pair of notes (Fig. 2).

#### Procedure

Conditions. There were separate sessions for the Joint Action (JA) and the Non-Interactive (Non-Int) tasks, which were presented in counterbalanced order between the participants. During the two tasks, identical stimuli were presented and the participants alternated with their partner in generating the notes. The conditions differed only for task instructions: in the JA condition the color cue (red, orange, blue or light blue) indicated which of the four four-note melodies the participant had to play *together with* their partner, in alternating turns of playing one note each (i.e., participants played two of the four notes in turn with their partner while remembering the full melody). In the Non-Int condition, the color cue (yellow, green, pink or violet) was associated with one of four pairs of notes that the participants had to play in two consecutive trials *independent of* the notes their partner was playing. For instance, the color cue could specify: JA condition, red melody C-C-G-G, orange melody C-G-C-G, blue melody G-G-C-C, light-blue melody G-C-G-C; Non-Int condition, yellow pair C-G; green pair C-C; pink pair G-C; violet pair G-G (the association between colors and melodies/pairs of notes was counterbalanced between participants). Thus, all color cues conveyed the same amount of information the participant needed to perform the task in two consecutive trials: in the example given above, both the red and the yellow cues indicated that the participants had to first play a C and then a G note. Importantly, however, the color cue in the JA condition also indicated the note that the partner would play, whereas the partner’s action was irrelevant in the Non-Int condition and therefore not indicated.

Experiment phases. Each JA/Non-Int session was divided into two phases: a Learning phase and a Test phase. During the *Learning phase* (about 20 min) the participants learned the association between a color cue and a melody (JA condition) or pair of notes (Non-Int condition) (see the Supplementary Information for more details on the Learning Phase). Only participants who successfully completed the Learning phase (threshold 80% of accuracy) could move on to the Test phase. No participant was excluded according to this criterion.

During the *Test phase*, the participants took turns with their virtual partner to complete the task. The Test phase of each JA/Non-Int session comprised 128 trials, as it included 16 repetitions of each four-note melody (JA) or pair of notes (Non-Int), each of which composed two trials. The session was divided into two blocks (64 trials each) with a 30 s break in between. Within each block, the order of melodies (JA) or pairs of notes (Non-Int) was pseudorandomized so that each melody/pair could not be consecutively repeated more than twice. The instructions were set up so that participants played either a G or a C note 50% of the time, and the combination of the participant’s and their partner’s actions was congruent or incongruent in 50% of the trials. Overall, the participants performed 32 trials per condition. Stimuli presentation and randomization were controlled by E-Prime2 software (Psychology Software Tools Inc.).

Trial timeline of the Test phase. A “trial” was counted as each time a participant performed a pointing or grasping action with the cube: thus, each musical sequence (both the four-note melodies in the JA condition and the pairs of notes in the Non-Int condition) consisted of two consecutive trials. Figure 2 illustrates the trial timeline, which was identical in the JA and Non-Int conditions. For each trial, the partner always took the first turn. Each trial started with the image of a fixation cross (400 ms) displayed on the monitor, followed by an image of the partner in the starting position (200 ms plus a variable stimulus onset asynchrony (SOA) ranging from 100 to 700 ms), then in the implied-motion position (duration 50% times 100/200 ms), and then in the final position, which was presented synchronously with the partner’s note. The image showing the partner’s final position also included the color cue indicating which melody (JA) or pairs of notes (Non-Int) the participant had to play. The partner’s note constituted the GO signal for the participants to release the start button and play their note. The correct note would be played if the response was correct, otherwise an error signal would sound. The participants were told to complete the task as quickly and correctly as possible. Importantly, and differently from Experiment 2, in Experiment 1 the association between the partner’s action (pressing the top or the side buttons) and the ensuing note (C or G note) was always identical to the participant’s. The participants familiarized themselves with the task and the apparatus in an 8-trial practice block before starting each JA/Non-Int Test phase.

Trial-type. Since the color cues, which corresponded to a melody or pair of notes, directed participants on what to do in two consecutive trials and appeared at the end of the partner’s first move, each melody or pair of notes effectively contained two trials: Trial-type1 (corresponding to the first half of the melody or pair of notes) when the participants observed their partner’s actions before seeing the color cue, and Trial-type2 (corresponding to the second half of the melody or pair of notes) when the participants had already seen (in the preceding Trial-type1) the cue and already knew what to do before observing their partner’s action (Fig. 2).

#### Data handling and design

We measured Accuracy (ACC), i.e., the proportion of correct responses over non-excluded trials, and Response Times (RTs), i.e., the time delay between the go signal and the instant the participant pressed a button measured in correct trials only. We also measured reaction times, i.e., the time delay between the go signal and the instant the participant released the start button, to exclude from the analysis of both ACC and RTs the trials in which participants made a false start (overall, 1.72 ± 2.20% of the trials in JA condition, 2.20 ± 2.81 trials per participant, and 2.19 ± 3.73% of the trials in Non-Int condition, 2.80 ± 4.77 trials per participant).

We calculated the individual mean ACC and RTs for each condition, excluding from the analysis of RTs any outlier values that fell 2.5 SDs above or below the mean for each experimental condition (average percentage of outlier trials 3.03 ± 1.22%, 3.88 ± 1.56 trials per participant in the JA condition, and 2.81 ± 1.26%, 3.60 ± 1.61 trials per participant in the Non-Int condition). Moreover, we excluded from the analysis any participant with a grand mean RT that fell 2.5 SDs above or below the group mean RT (no participant was excluded according to this criterion). To analyze a dependent variable that appropriately weighs the impact of speed and accuracy and thus captures participant performance as a whole^23^, we calculated the Inverse Efficiency Score (IES), i.e., the Response Times (RTs)/ACC ratio. IES data were normally distributed according to Shapiro-Wilk and Kolmogorov-Smirnov tests, and a 2 × 2 × 2 repeated-measures ANOVA was performed with Task (JA vs. Non-Int) x Trial-type (Trial-type1 vs. Trial-type2) x Congruence of Actions (Congruent vs. Incongruent) as within-subject factors. Raw ACC and RTs data per condition are reported in the Supplementary Information (see Supplementary Table S1). All tests of significance were based upon an α level of 0.05. When appropriate, post-hoc tests were performed using Bonferroni correction.

We expected a Task x Congruence interaction, indicating that visuomotor interference (indexed by an IES-Incongruent > IES-Congruent difference) emerges in the Non-Int but not in the JA condition. To show that a possible lack of significant effect of Congruence in JA indeed provides evidence in favor of a null effect of Congruence in this condition, we applied Bayesian statistical analysis, as implemented in JASP^52^. The rationale for this analysis is that the Bayes Factor (BF_10_) is a statistical metric that quantifies the strength of evidence that the data provide in favor of the alternative hypothesis relative to the null hypothesis: a BF_10_ higher than 3 indicates substantial evidence supporting the alternative hypothesis, whereas a BF_10_ lower than 0.3 indicates substantial evidence supporting the null hypothesis^53^. We calculated the individual mean IES in the Incongruent and Congruent conditions, separately for the Non-Int and the JA tasks, and ran two one-tailed Bayesian paired t-tests to test for the presence of an Incongruent > Congruent difference, which would suggest the presence of visuomotor interference.

### Experiment 2

#### Participants

A total of 25 participants took part in the study (5 men, age range 20–29 years, mean 23.5 ± 2.1). All participants were right-handed as confirmed by the Standard Handedness Inventory^51^, reported normal or corrected-to-normal vision, and were naive as to the purpose of the experiment.

#### Stimuli and apparatus

Stimuli and apparatus were the same as in Experiment 1.

#### Procedure

The procedure was similar to Experiment 1, except that in Experiment 2 each JA/Non-Int session was divided into three rather than two phases: (i) the Learning phase was identical to Experiment 1; (ii) the Test phase was similar to Experiment 1 (see below); (iii) and a Check phase was added to verify the impact on participant performance of Congruence of Notes independent of the physical congruence between the partner’s and the participant’s actions (in this phase, the partner was not shown on the screen, see the Supplementary Information and Supplementary Fig. S1 for more details). As in Experiment 1, only those participants who successfully completed the Learning phase (threshold 80% of accuracy) could move on to the Test phase. One participant was excluded according to this criterion. The trial timeline of the Test phase was identical to Experiment 1.

In the Test phase, each JA/Non-Int session included 256 trials, divided into 16 mini-blocks of 16 trials each. In each mini-block, each of the four melodies (JA) or pairs of notes (Non-Int) was presented twice. An additional factor included in this Experiment was that the action-note association was reversed for the partner in 50% of the mini-blocks but never changed for the participants. For the Coherent-Association trials, the participants interacted with a partner whose response-box worked identically to the participant’s (i.e., pointing to the top button generated a G note and grasping the side buttons generated a C note), whereas for the Reversed-Association trials the participants interacted with a partner whose response-box worked opposite theirs (i.e., pointing to the top button generated a C note and grasping the side buttons generated a G note). The order of Coherent/Reversed association was pseudorandomized so that the same Coherent/Reversed mini-block could not occur more than twice consecutively. As in Experiment 1, the instructions were set up so that the participants played either a G or C note 50% of the time, and the combination between the participant’s and partner’s action was congruent or incongruent in 50% of the trials. Overall, participants performed 32 trials per condition.

#### Data handling and design

Dependent variables and data handling were identical to those in Experiment 1. In addition to ACC and RTs we also measured reaction times to exclude from the analyses the trials in which participants made a false start (0.73 ± 1.91% of the trials in the JA condition, 1.88 ± 4.88 trials per participant, and 1.22 ± 2.75% of the trials in the Non-Int condition, 3.13 ± 7.03 trials per participant).

We calculated the individual mean ACC and RTs for each condition, excluding from the analysis of RTs any outlier values that fell 2.5 SDs above or below the mean for each experimental condition (average percentage of outlier trials 2.69 ± 1.17%, 6.88 ± 2.98 trials per participant in the JA condition, and 2.93 ± 0.82%, 7.50 ± 2.11 trials per participant in the Non-Int condition). We excluded from the analysis any participant with a grand mean RT that fell 2.5 SDs above or below the group mean (one participant was excluded by this criterion, and one participant did not pass the Learning phase; final sample N = 23). Finally, we calculated IES for each condition and performed 2 × 2 × 2 × 2 repeated-measures ANOVA with Task (JA vs. Non-Int) x Trial-type (Trial-type1 vs. Trial-type2) x Congruence of Actions (Congruent vs. Incongruent) x Action-Note Association (Coherent vs. Reversed) as within-subject factors (Fig. 4). Raw ACC and RTs data per condition are reported in the Supplementary Information (see Supplementary Tables S2 and S3). All tests of significance were based upon an α level of 0.05. When appropriate, post-hoc tests were performed using Bonferroni correction.

We expected a Task x Congruence and Task x Association interaction, indicating that (i) visuomotor interference emerges in the Non-Int but not in the JA condition, and (ii) a Reversed Association impairs performance in the JA but not in the Non-Int condition. As done in Experiment 1, Bayesian statistics was applied to ensure that the data provided evidence in favor of the null hypothesis with regard to the effect of Congruence in the JA task and the effect of Association in the Non-Int task. We calculated the individual mean IES in the Incongruent and Congruent conditions and in the Coherent and Reversed Association conditions separately for the Non-Int and JA tasks. We then performed four one-tailed Bayesian paired t-tests to test for the presence of an Incongruent > Congruent difference and for the presence of a Reversed > Coherent Association difference, separately for the JA and the Non-Int tasks.

### Data Availability

All data are made available by authors upon request.

## Electronic supplementary material

Supplementary Results and Supplementary Methods

Supplementary Video 1 Example of coherent association (G-C-G-C sequence)

Supplementary Video 2 Example of reversed association (C-G-G-C sequence)
