# Supplementary material for: Evidence for a dyadic motor plan in joint action
Source: Sci Rep. 2018 Mar 22;8:5027. doi: 10.1038/s41598-018-23275-9 (PMC5864721; doi:10.1038/s41598-018-23275-9)
Supplement: Supplementary file 1 — Supplementary Results and Supplementary Methods [file 41598_2018_23275_MOESM1_ESM.docx]

**Supplementary Information**

**Evidence for a dyadic motor plan in joint action**

Lucia Maria Sacheli^1,2*^, Elisa Arcangeli^1^, Eraldo Paulesu^1,2^

1. Department of Psychology and Milan Center for Neuroscience (NeuroMi), University of Milano-Bicocca, 20126 Milan, Italy.
2. IRCCS Istituto Ortopedico Galeazzi, 20161 Milan, Italy

* Corresponding author:

Lucia Maria Sacheli

Piazza dell’Ateneo Nuovo 1, 20126 Milan, Italy

+39 02 64483776; [lucia.sacheli@unimib.it](mailto:lucia.sacheli@unimib.it)

**Supplementary Results**

***Experiment 1 – Physical congruence of action does not modulate behavior in JA***

The table below summarizes raw Accuracy (Acc) and Response Times (RTs) data per condition.

**Supplementary Table S1**. Data collected during Experiment 1.

| **Accuracy** | | | | | | | | |
| --- | --- | --- | --- | --- | --- | --- | --- | --- |
|  | **JA-T1-C** | **JA-T1-In** | **JA-T2-C** | **JA-T2-In** | **NI-T1-C** | **NI-T1-In** | **NI-T2-C** | **NI-T2-In** |
| ***Median*** | 1.00 | 1.00 | 1.00 | 1.00 | 0.97 | 1.00 | 1.00 | 1.00 |
| ***Range*** | 0.69 – 1.00 | 0.88 – 1.00 | 0.84 – 1.00 | 0.93 – 1.00 | 0.94 – 1.00 | 0.75 – 1.00 | 0-97 – 1.00 | 0.94 - 1.00 |
| **Response Times** | | | | | | | | |
|  | **JA-T1-C** | **JA-T1-In** | **JA-T2-C** | **JA-T2-In** | **NI-T1-C** | **NI-T1-In** | **NI-T2-C** | **NI-T2-In** |
| ***Mean*** | 975.78 | 976.67 | 796.20 | 771.76 | 996.43 | 1027.82 | 784.48 | 801.02 |
| ***SD*** | 167.71 | 149.57 | 145.98 | 130.21 | 145.30 | 162.93 | 134.45 | 125.75 |

JA = Joint Action task, NI = Non-Interactive task; T1 = Trial-type1, T2 = Trial-type2; C = Congruent, In = Incongruent.

***Experiment 2 – Prediction of action effects uniquely takes place in interactive contexts***

The tables below (Supplementary Tables S2 and S3) summarize raw Accuracy (Acc) and Response Times (RTs) data per condition, separately for data collected during Trial-type1 and Trial-type2 trials.

**Supplementary Table S2**. Data collected during Trial-type1 in Experiment 2.

| **Accuracy – Trial-type1** | | | | | | | | |
| --- | --- | --- | --- | --- | --- | --- | --- | --- |
|  | **JA-Co-C** | **JA-Co-In** | **JA-Rev-C** | **JA-Rev-In** | **NI-Co-C** | **NI-Co-In** | **NI-Rev-C** | **NI-Rev-In** |
| ***Median*** | 0.97 | 0.97 | 0.94 | 0.97 | 1.00 | 1.00 | 1.00 | 1.00 |
| ***Range*** | 0.85 – 1.00 | 0.88 – 1.00 | 0.47 – 1.00 | 0.75 – 1.00 | 0.94 - 1.00 | 0.91 - 1.00 | 0.90 - 1.00 | 0.87 - 1.00 |
| **Response Times – Trial-type1** | | | | | | | | |
|  | **JA-Co-C** | **JA-Co-In** | **JA-Rev-C** | **JA-Rev-In** | **NI-Co-C** | **NI-Co-In** | **NI-Rev-C** | **NI-Rev-In** |
| ***Mean*** | 1091.93 | 1144.82 | 1320.33 | 1191.46 | 961.56 | 976.67 | 951.30 | 981.63 |
| ***SD*** | 290.45 | 285.21 | 322.03 | 335.67 | 131.42 | 131.02 | 132.74 | 141.58 |

JA = Joint Action task, NI = Non-Interactive task; Co = Coherent action-note association, Rev = Reversed action-note association; C = Congruent, In = Incongruent.

**Supplementary Table S3**. Data collected during Trial-type2 in Experiment 2.

| **Accuracy – Trial-type2** | | | | | | | | |
| --- | --- | --- | --- | --- | --- | --- | --- | --- |
|  | **JA-Co-C** | **JA-Co-In** | **JA-Rev-C** | **JA-Rev-In** | **NI-Co-C** | **NI-Co-In** | **NI-Rev-C** | **NI-Rev-In** |
| ***Median*** | 1.00 | 1.00 | 1.00 | 1.00 | 1.00 | 1.00 | 1.00 | 1.00 |
| ***Range*** | 0.88 – 1.00 | 0.90 – 1.00 | 0.90 – 1.00 | 0.81 – 1.00 | 0.96 – 1.00 | 0.93 – 1.00 | 0.93 – 1.00 | 0.91 – 1.00 |
| **Response Times – Trial-type2** | | | | | | | | |
|  | **JA-Co-C** | **JA-Co-In** | **JA-Rev-C** | **JA-Rev-In** | **NI-Co-C** | **NI-Co-In** | **NI-Rev-C** | **NI-Rev-In** |
| ***Mean*** | 813.08 | 800.93 | 861.95 | 868.06 | 782.96 | 804.09 | 792.09 | 796.37 |
| ***SD*** | 155.77 | 140.50 | 156.86 | 159.56 | 121.72 | 139.94 | 114.90 | 133.40 |

JA = Joint Action task, NI = Non-Interactive task; Co = Coherent action-note association, Rev = Reversed action-note association; C = Congruent, In = Incongruent.

***Results on data collected during Trial-type1 of the JA condition in Experiment 2***

The follow-up ANOVA performed on data collected during Trial-type1 of the JA condition showed a significant main effect of Association (F(1,22) = 22.92, *p* < .001, η_p_^2^ = .51), indicating that participant performance was generally less efficient (i.e., IES were higher) when interacting with a partner who played with a Reversed Action-Note Association, in line with the results that emerged from the analysis of Trial-type2 data. Yet, the ANOVA also showed a main effect of physical Congruence of Action (F(1,22) = 5.49, *p* = .03, η_p_^2^ = .20), indicating that performance was generally less efficient (i.e., IES were higher) in Congruent as compared to Incongruent trials, thus showing a pattern opposite to the one that would indicate visuomotor interference (and that would be predicted by the Dual-Route hypothesis). Finally, the ANOVA showed a significant Association x Congruence interaction (F(1,22) = 6.67, *p* = .02, η_p_^2^ = .23): it indicates that performance in Reversed-Association trials was less efficient than in Coherent-Association trials only in the Congruent (*p*_corr_ = .003) but not in the Incongruent (*p*_corr_ = .25) condition. As shown by a control analysis, the strengths of this unexpected Association x Congruence interaction effect depended on individuals’ sensitivity to the congruence of note as measured during the Check phase (see below). Importantly, however, performance in Congruent vs. Incongruent trials never differed, either in Coherent (*p*_corr_ = .7) or Reversed (*p*_corr_ = .1) Association trials, in line with the results that emerged from the ANOVA performed on Trial-type2 data of the JA condition and replicating results of Experiment 1 (see main text).

***Performance in the Check phase and control analysis on individuals’ Sensitivity to Congruence of Note (Experiment 2).***

The ANOVA on IES recorded during the Check phase showed a significant main effect of Task (F(1,22) = 7.06, *p* = .01, η_p_^2^ = .24) and a significant main effect of Trial-type (F(1,22) = 117.83, *p* < .001, η_p_^2^ = .84) indicating that, overall, participant performance was more efficient in the Non-Int than the JA condition and in Trial-type2 than Trial-type1. The ANOVA also showed significant Task x Trial-type (F(1,22) = 9.36, *p* = .006, η_p_^2^ = .30), Trial-type x Congruence (F(1,22) = 5.94, *p* = .02, η_p_^2^ = .21), and Task x Trial-type x Congruence (F(1,22) = 4.76, *p* = .04, η_p_^2^ = .18) interactions. We then performed two follow-up ANOVAs separately per each Task. The ANOVA on the Non-Int condition only showed a significant main effect of Trial-type (F(1, 22) = 94.18, *p* < .001, η_p_^2^ = .81) indicating that participant performance was more efficient in Trial-type2 than Trial-type1. On the contrary, the ANOVA on the JA condition showed a significant main effect of Trial-type (F(1, 22) = 71.41, *p* < .001, η_p_^2^ = .76) but also a significant Trial-type x Congruence interaction (F(1,22) = 5.90, *p* = .02, η_p_^2^ = .21), which showed that, in Trial-type1, performance in Congruent trials tended to be more efficient than in Incongruent ones (*p*_corr_ = .054). This suggests that, in Trial-type1 and only in the JA task, at least some of the participants tended to be influenced by the Congruence of Note and to be more efficient when the note that they had to play was the same as the one just played by the computer.

The pattern described above, showing a Trial-type x Congruence interaction in the JA task only, somehow paralleled the one that emerged from the analysis of participant performance during the Test phase of the JA task: we thus exploratively tested whether there was a relation between participants’ sensitivity to the Congruence of Note (as measured during the Check phase) and their decay in performance during the Test phase of the JA task in the Congruent-Reversed Association condition. Indeed, it is worth noting that in the Congruent-Reversed Association condition participants, although performing a movement that is congruent with the one observed in the partner, have to play a note that is incongruent with the one that they have just heard: thus, their decay in performance might at least partially depend on their sensitivity to the congruence of note (i.e., on their tendency to be impaired when they have to play an incongruent note). In order to test this hypothesis, we performed a control analysis by using the index measuring the individuals’ sensitivity to the congruence of note (“Congruence-Sensitivity Index” = IES-Incongruent minus IES-Congruent, recorded during the Check phase of the JA task in Trial-type1) as a continuous predictor in a GLM having as dependent variable the IES recorded during the Test phase of the JA task (Trial-type1 only): the design of such analysis included Association (Coherent / Reversed) and Congruence of Actions (Congruent / Incongruent) as within-subject factors and the above-mentioned Congruence-Sensitivity Index as continuous predictor.

The GLM showed a significant main effect of Congruence-Sensitivity Index and Association, and significant Congruence-Sensitivity Index x Congruence and Congruence-Sensitivity Index x Association x Congruence interactions (see Table S4).

**Supplementary Table S4**. Results that emerged from the GLM on data collected during the Test phase (JA task, Trial-type1 only) and having the Congruence-Sensitivity Index (obtained from data recorded during the Check phase) as continuous predictor.

| **GLM having individuals’ sensitivity to Congruence of Note as continuous Predictor** | | | | |
| --- | --- | --- | --- | --- |
| **Effect** | **F** | **Df** | **p** | **η_p_2** |
| Main effect of Congruence-Sensitivity Index | 4.67 | 1,22 | .042 | .18 |
| Main effect of Association | 14.84 | 1,22 | < .001 | .41 |
| Congruence-Sensitivity Index x Congruence | 6.51 | 1,22 | .019 | .24 |
| Congruence-Sensitivity Index x Association x Congruence | 10.07 | 1,22 | .005 | .32 |

These results suggest that the unexpected Association x Congruence interaction that emerged from the analysis of the Test phase in JA (Trial-type1 only) was indeed explained by individuals’ sensitivity to the congruence of notes, because the Congruence-Sensitivity Index moderated the Association x Congruence interaction. In order to interpret the direction of such an effect, we calculated an index of the effect of interest [IES in the Test phase of the JA task in Trial-type1-Reversed Association: “IES-Congruent minus IES-Incongruent”] and correlated it with the Congruence-Sensitivity Index. The results showed a significant correlation between the two measures: *r* = .55, *p* < .007, corrected in *r* = .43, *p* = .047 when one outlier value (according to the criterion Cook distance > 1) was excluded from the analysis.

These results suggest that the unexpected interaction effect between Association and Congruence of Action in Trial-type1 of the JA task (that emerged from the analysis of the Test phase) was actually due to participants’ sensitivity to Congruence of Note: the more participants showed the tendency to be impaired when they had to play an incongruent note in Trial-type1, the more they were impaired in the Congruent as compared to the Incongruent condition during Reversed Association trials. Importantly, however, these results are in line with the take-home message of the study: participant performance in the JA condition never showed an effect of Congruence of Actions, and was influenced by action effects (e.g., by congruence of notes), while the latter did not influence performance in the perceptually-matched Non-Int condition.

***Experiment 3 and 4 – Replication of Experiment 2 results***

**Experiment 3** (N=16, age 23.56 ± 3.12, f = 9) aimed to replicate the results that emerged in the Non-Int condition in Experiment 2. The set-up, experimental stimuli and trial time-line were identical to those described in the main text for Experiment 1 and 2. Yet, in this experiment a color cue (yellow or green) directly instructed participants on what single movement to perform (grasping vs. pointing) independently from the note (color-movement association was counterbalanced between subjects). Thus, the factor Trial-type was not present, as each color cue instructed participants on what to do at each single trial, so that each trial was independent from the previous one. This was done in order to test whether the lack of an effect of Association would be replicated in a task more similar to a classic visuomotor interference paradigm. Analyses were based on a 2x2 repeated-measure design having Congruence of Actions (Congruent vs. Incongruent) x Partner’s Action-Note Association (Coherent vs. Reversed) as within-subject factors.

Results were in line with those that emerged in Experiment2: the ANOVA only showed a significant main effect of Congruence of Actions (F(1, 15) = 7.69, *p* = .014, η_p_^2^ = .34) and no main effect or interaction with the factor Association (all *p*s > .2).

**Experiment 4** (N=16, age 25.50 ± 3.39, f = 11) was aimed to replicate the results that emerged in the JA condition in Experiment 2. The set-up, experimental stimuli and trial time-line were identical to those described in the main text for Experiment 1 and 2. The only difference regarded the structure of the four possible melodies, which slightly differ from the one described in the main text and was as follow:

- Randomization A: G-C-C-G, G-G-G-C, C-G-C-C, C-C-G-G;
- Randomization B: C-G-G-C, C-C-C-G, G-C-G-G, G-G-C-C.

This was done in order to ensure that the results that emerged from the analysis of Experiment 2 were not due to the specific structure of the melodies that participants had to play. Half of the sample learnt the melodies from Randomization A and half did so from Randomization B, and the association between colors and melodies was counterbalanced between participants. Analyses were based on a 2x2x2 repeated-measure design having Trial-type (Trial-type1 vs. Trial-type2) x Congruence of Actions (Congruent vs. Incongruent) x Partner’s Action-Note Association (Coherent vs. Reversed) as within-subject factors.

Results were in line with those that emerged in Experiment 2: the ANOVA showed a significant main effect of Association (F(1, 15) = 49.22, *p* < .001, η_p_^2^ = .77) indicating that performance in the Reversed Association was less efficient than in the Coherent Association; the analysis also showed a main effect of Trial-type (F(1, 15) = 17.20, *p* < .001, η_p_^2^ = .53), indicating that performance in Trial-type1 was less efficient than in Trial-type2, and a main effect of Congruence of Actions (F(1, 15) = 15.76, *p* < .001, η_p_^2^ = .51), indicating that performance in Congruent trials was less efficient than in the Incongruent trials, thus showing a pattern that was opposite to the one indicating visuomotor interference effects. All other significant interactions up to the triple Trial-type x Congruence x Association interaction (F(1, 15) = 13.58, *p* = .002, η_p_^2^ = .47) were also significant.

In order to make the triple interaction easier to interpret we performed two follow-up ANOVAs separately for each Trial-type. The ANOVA on the **Trial-type2** data showed a main effect of Association (F(1, 15) = 20.80, *p* < .001, η_p_^2^ = .58) indicating that performance in the Reversed Association was less efficient than in the Coherent Association, but also a main effect of Congruence of Actions (F(1, 15) = 8.27, *p* = .012, η_p_^2^ = .35), indicating that performance in Congruent trials was less efficient than in the Incongruent trials, thus showing a pattern that was opposite to the one indicating visuomotor interference effects, in line with Experiment 2. Finally, the ANOVA showed a significant Congruence x Association interaction (F(1, 15) = 6.28, *p* = .024, η_p_^2^ = .29): post-hoc tests (Bonferroni-corrected) showed that participants were less efficient in both Reversed-Association conditions as compared to Coherent-Association conditions (all *p*s*_corr_* < .02); the Congruent condition was also less efficient that the Incongruent condition in Reversed-Association trials (*p_corr_* = .02). The ANOVA on the **Trial-type1** data showed a main effect of Association (F(1, 15) = 11.09, *p* = .005, η_p_^2^ = .42) indicating that performance in the Reversed Association was less efficient than in the Coherent Association, but also a main effect of Congruence of Actions (F(1, 15) = 13.42, *p* = .002, η_p_^2^ = .47), indicating that performance in Congruent trials was less efficient than in Incongruent trials, thus showing a pattern that was opposite to the one indicating visuomotor interference effects, in line with Experiment 2. Finally, the ANOVA showed a significant Congruence x Association interaction (F(1, 15) = 17.13, *p* < .001, η_p_^2^ = .53): post-hoc tests (Bonferroni-corrected) showed that participants were less efficient in Congruent as compared to Incongruent trials in the Reversed-Association condition (*p_corr_* = .002), and that performance in the Congruent-Reversed-Association condition was also less efficient that in the Congruent-Coherent-Association condition (*p_corr_* = .003).

Overall, these results replicated the ones that emerged from the analysis of the JA condition in Experiment 2 by showing that, in an interactive context, participant performance shows no sign of visuomotor interference effects while it shows a tendency to predict the effects of the partner’s action from observation.

**Supplementary Methods**

Both in Experiment 1 and 2, the Test phase described in the main text was preceded by a Learning phase aimed to teach participants the association between each color cue and the respective melody or pair of notes. In Experiment 2, the Test phase was also followed by a Check phase aimed to test participant performance during a task that was identical to the one performed during the Test phase expect for the visual information provided by the observation of the partner’s action, which was removed during the Check phase.

***Learning phase.*** Each JA / Non-Int Test phase was preceded by the corresponding Learning phase.

In both Experiment 1 and 2, the structure of participants’ melodies and pairs of notes was identical and as follows (association between colors and melodies or pairs of notes was counterbalanced between participants):

- Melodies (JA): (i) C-C-G-G, (ii) C-G-C-G, (iii) G-G-C-C, (iv) G-C-G-C;
- Pairs of Notes (Non-Int): (i) C-G, (ii) G-G, (iii) G-C, (iv) C-C.

Each Learning phase was divided into two sessions. During the *first learning session*, participants heard each melody / pair of notes while being concurrently presented with the corresponding color cue, and they were asked to immediately reproduce it. Each melody / pair of notes was consecutively presented until participants correctly reproduced it five consecutive times. Afterwards, participants were randomly presented the color cues and were required to produce the corresponding melody / pair of notes. The color cue corresponding to each melody / pair of notes was presented 10 times, and participants had to correctly produce each melody / pair of notes at least 8 times (corresponding to an accuracy threshold equal to 80%) in order to move forward to the next learning session, otherwise they were asked to repeat the first session. No participant repeated the first session more than twice.

During the *second learning session*, participants performed a recognition task: they heard each melody / pair of notes 10 times (random presentation) and had to identify the corresponding color cue. Participants had to correctly identify each melody / pair of notes 8 out of 10 times (corresponding to an accuracy threshold equal to 80%) in order to move forward to the Test phase, otherwise they were asked to repeat the recognition task. Only one participant in Experiment 2 did not successfully pass the threshold after two repetitions of the recognition task, and was thus excluded from the study.

***Check phase.*** During the Check phase, participants had to play the JA / Non-Int task in turn-taking with the computer while only hearing the partner’s notes, as no virtual partner was shown on the screen. Thus, the trial timeline was identical to the one of the Test phase described in the main text (see Figure 2 in the main text), yet after the fixation-cross participants only saw the color cue and concurrently heard the partner’s first note, without seeing his movement, and had to correctly respond by playing the second note of the melody (JA) or the first of the pair of notes. See Supplementary Figure S1. If participants performed the wrong response, an error sound was played, as in the Test phase. Participants completed 96 trials per session, including 24 trials per condition (Trial-type1/Trial-type2 x Congruent/Incongruent). Please note that here Congruence (Congruent/Incongruent) refers to the combination between the participant’s note and that of the computer, and not to congruence of actions (because no partner’s action was shown). As in the Test phase, we calculated participants’ Accuracy (ACC), reaction times, i.e., the time-delay between the go-signal and the instant when participants released the start button (to exclude from the analysis the trials in which participants made a false start), and Response Times (RTs, i.e., the time-delay between the go-signal and instant when participants pressed the response buttons, measured in correct trials only). Performance was indexed by Inverse Efficiency Scores, i.e., the RTs/ACC ratio, which were entered a 2x2x2 ANOVA with Task (JA/Non-Int) x Trial-type (Trial-type1/Trial-type2) x Congruence of Notes (Congruent/Incongruent) as within-subject factors.


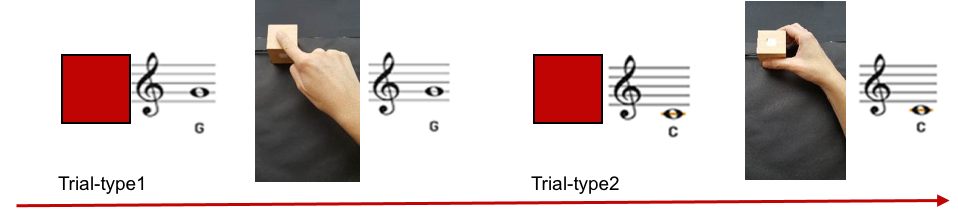


**Supplementary Figure S1.** The trial timeline of the Check phase, which was identical in the JA and Non-Int sessions.
